# Reply to “Increased Prevalence of Breast and All-cause Cancer in Female Orthopedic Surgeons”: Alcohol Consumption as a Possible Contributing Factor

**DOI:** 10.5435/JAAOSGlobal-D-22-00272

**Published:** 2023-09-06

**Authors:** Elizabeth K. Farkouh, Ian M. Michel, Keshav Poudel, Norman Giesbrecht, Timothy S. Naimi

**Affiliations:** From the Mayo Clinic, Rochester, MN (Ms. Farkouh, Mr. Michel, Mr. Poudel), Institute for Mental Health Policy Research, Centre for Addiction and Mental Health, Toronto, ON, Canada (Dr. Giesbrecht), Dalla Lana School of Public Health, University of Toronto, Toronto, ON, Canada (Dr. Goesbrecht), Canadian Institute for Substance Use Research, University of Victoria, Victoria, BC, Canada (Dr. Naimi).

*To the Editor*, We read with great interest the article entitled “Increased Prevalence of Breast and All-cause Cancer in Female Orthopedic Surgeons” by Chou et al.^1^ (2022). The study, which used a survey-based sample of 672 female orthopaedic surgeons, found a 1.89 and 3.97-fold increased prevalence of all-cause cancer and breast cancer, respectively, among female orthopaedic surgeons compared with matched US women from the general population.

The results raise notable concern as to the reasons for the increased prevalence of breast and other cancers. In their discussion, the authors propose reasons including higher socioeconomic status, delayed age of childbearing, and occupational factors. The authors also note that poor-fitting lead protection garments among female trainees could lead to increased radiation exposure to breast tissues and subsequently advocate for change in this area.

While we agree with recommendations to have proper-fitting lead protection garments for all surgeons and believe that additional efforts toward equity are of paramount importance in medicine, the claim that radiation exposure may be contributing to the higher risk of breast and other cancers among female orthopaedic surgeons was generally not supported by the results of the study. The authors found no notable difference in the prevalence of cancer by individual subspecialty, use of fluoroscopy, and use of polymethyl methacrylate (PMMA), nor use of protective shielding. In fact, the authors found that cancer prevalence was markedly *lower* among surgeons in subspecialties likely to have greater radiation exposure. In addition, the authors did not find an increased breast cancer risk by radiation exposure group or specialty type.

The authors also noted that, compared with general population–based female samples, female orthopaedic surgeons in their sample were more likely to have breast cancer protective factors such as being of a younger age, having a normal body mass index, having never smoked, being White, and having had less exposure to hormone replacement therapy. The authors stated, “Despite these protective factors, female orthopedic surgeons still reported a higher prevalence of cancer compared with matched US women. Additional investigation is needed to further delineate these variables and their correlation with cancer risk.”^1^

Despite many protective factors being present in their sample, the authors neglected to discuss the markedly increased prevalence of high-risk alcohol consumption among their sample. Alcohol is a class 1 carcinogen, and there is notable evidence of alcohol's contribution to breast and other cancer risk.^2^ Alcohol consumption as low as three drinks per week has been associated with a 15% increased risk of breast cancer.^3^

Despite not being reported in the body of the study, Chou et al. (2022) collected data on alcohol consumption among their sample of female orthopaedic surgeons that were reported in Appendix Table A2. Their data showed that female orthopaedic surgeons were consuming alcohol at a level considered to markedly increase risk of breast cancer (3 or more drinks per week^4^) at approximately 3.7 times the rate of women in the general population (34.7% vs 9.5%). In the surgeon group, 25.3% of women were consuming 3 to 5 drinks per week compared with 8.6% of the general population and 9.4% of surgeons were drinking six or more drinks per week compared with 0.9% of the general population.

In addition, it is very likely that with on-call and other responsibilities, the surgeons who consume six or more drinks per week may have been consuming several drinks on one occasion (i.e., possible binge drinking). Such drinking behavior markedly increases the risk of breast cancer.^5^

Despite the notable concern for high-risk alcohol consumption among their sample, Chou et al. (2022) did not analyze the rate of cancer diagnosis in relation to levels of alcohol intake. We think such an association is very likely, and the lack of analysis represents a missed opportunity for identification of another potential cause of the increased cancer rate in this population.

Beyond increased cancer risk, the markedly increased levels of high-risk alcohol consumption raise concerns about the psychosocial well-being of many female surgeons who may be consuming alcohol to cope with stress. This group is drinking more with less free time than the general population. A national survey found that 25.6% of female surgeons engaged in patterns of alcohol use concerning for “abuse” or dependence and that this group was more likely to report burnout, depression, and a major medical error in the previous 3 months.^6^ This raises concerns about surgeon well-being beyond cancer risk. As per the report by Chou et al. (2022), the percentage of female orthopaedic surgeons is increasing. It is, therefore, imperative that efforts focus not only on hazards encountered in the workplace but also on social and emotional aspects of wellness.

In conclusion, the increased rates of cancer and breast cancer among female orthopaedic surgeons reported by Chou et al. (2022) raise notable concerns. Although the authors focused mainly on radiation exposure as a contributory cause, their evidence did not generally support that association. While we agree that radiation protection is an actionable change that should be pursued, we think that the evidence presented by the authors suggests a possible role of alcohol consumption for the increased cancer rate among female surgeons. Additional research is needed to determine contributory causes of increased cancer prevalence so that evidence-based interventions can be developed that will lead to reduced cancer risk and ultimately cancer prevention. Finally, the increased prevalence of high-risk alcohol consumption among this population warrants additional investigation and the development of clinical and policy-based interventions to address this key risk factor for negative health consequences.
